# Dr. Crichton, on a Note in Dr. Beddoes's "Contributions"

**Published:** 1799-10

**Authors:** Alexander Crichton

**Affiliations:** No. 15, Clifford-Street


VOL. II.] OCTOBER, 1799. [NO. VIII.

### To the Editors of the Medical and Physical Journal.

GENTLEMEN,

IN the "*Contributions*" lately published by Dr. BEDDOES, I observe that I have offended that gentleman to such a degree as to make him throw off the politeness of the scholar, and forget the language which one well-educated man has a right to expect from another. I am the more concerned at this, because I greatly suspect that, in justifying myself, I shall produce still more irritation than appears in the work alluded to; for I shall be under the necessity of proving by extracts from his former writings, either that he did not understand the language he employed at the time he wrote them—or that, with a deep, and wary penetration, he has given birth to different opinions on the same subject, to the end, that when one goes out of fashion, he may bring the others forward as a protest against it, if necessary.

I will now enter on my defence, but before I do so, let me state my arraignment.

The following note, bearing the genuine type of the meek spirit of Dr. Beddoes, is to be found at page 369 of his '*Contributions*:"

"Dr. CRICHTON ("*Mental Derangement*," 1—46,) says, 'There is scarce ' any treatment of consumption but has shewn equal, if not superior powers to a reduced atmosphere.' "The repose alone, which I have often known " follow the use of gases, seems to shew that this is a mistake. I have no-" where said, that occasional small respiration of gases and vapours have " cured, or promise to cure consumption. And where have they been " kept constantly applied to diseased lungs? Can Dr. C.'s learning supply " a satisfactory reference to facts of this nature? Dr. C. asserts (p. 35) " that I have adopted Dr. GIRTANNER's opinion concerning irritability. " This is false. In my earliest conjectures ("*Observ. on Calculus, &c.*" " p. 264), I have protested against this interpretation of my words; and

"Dr. C. ſhould have attended to what I have ſince written, ſince he choſe "to notice my opinions."——

Here I ſtop, Gentlemen, for it is this laſt part of the note which has cauſed me to trouble you with this letter.

I am charged with having given a falſe repreſentation of the Doctor's opinion concerning Irritability. The following are the words I have made uſe of, and which have given Dr. Beddoes offence:—"It may appear, "neverthelefs, to many, that oxygen is probably the principle on which "the irritability of bodies depends. This ſeems to be Dr. Beddoes's "opinion, as well as Dr. Girtanner's, and he (Dr. B.) makes it a ground "of argument in favour of his aërial method of treating phthiſis."

That it was natural to draw this concluſion, I will venture to aſſert, will be the opinion of every impartial man who has read the Doctor's "*Eſſays on Obeſity and Conſumption*," which are annexed to the "*Eſſay on Calculus*," to which he refers.

Dr. Girtanner thinks the ſolids obtain their oxygen from the blood during circulation. That Dr. B. thinks ſo alſo will appear *probable* from the following paſſages:—"Probably the ſolids, during circulation, more "than divide with the blood, its looſely attached oxygen; if they have a "ſuperior attraction, they will, as ſome of the conſtituent parts of the "blood itſelf do upon ſtanding, take the whole, and leave the blood dark-"coloured."—*Beddoes on Calculus, &c.*—p. 121.

Dr. Girtanner thinks that oxygen not only yields the ſtimulating quality to the blood, but that it is alſo the cauſe of irritability in the ſolids. That Dr. Beddoes appears to entertain, or rather appears to *have* entertained this notion, will probably be the opinion of many others beſides me, after they have read the following:—

At p. 137, "*Obſerv. on Calculus*," he makes an extract from a paper of Lavoisier's, in which that ingenious chemiſt and philoſopher relates the effects which oxygen gas produced on Guinea-pigs, that were made to live in it for a certain time; they died with evident marks of inflammation. Dr. Beddoes accounts for this effect in the following words, p. 138.—"The unuſual animal heat which muſt have been generated in theſe "experiments—*the ſtimulant power of the blood,* which, independently of "heat, oxygen confers upon the blood—*that irritability which it commu-"nicates to the ſolids*—all theſe cauſes might eaſily produce the inflam-"mation obſerved by Lavoiſier."

From the above, I was led to conclude that Dr. Beddoes really meant what he wrote; and I was the more confirmed in this opinion as I did not diſcover discover in any of his remarks on Dr. Girtanner's Essay, that he had entered a formal protest against Dr. Girtanner's opinion, although in the note which relates to me, in his "*Contributions*," he positively has done so.— I shall now insert the whole of the passage contained in the page to which he refers in the note, and in which he says he has protested against the opinion, that oxygen is the cause of irritability, which opinion, from the extracts above, I concluded him to possess.

"Attention," says Dr. Beddoes, p. 264, "is undoubtedly not less due "to the other elements of organized bodies; and if the importance of "oxygen seems to have been magnified in the foregoing observations, it "is only because we have few or no facts which afford a foundation for "reasoning concerning the connection of an excess or deficiency of hydro- "gen, or azote, with the functions of life: and yet much obscurity and "many difficulties must be expected to remain till we acquire the know- "ledge of such facts. This reflection should render us the more attentive "to the phenomena of life; for if we can but perceive enough to suggest "a new hypothesis, capable of being verified by experiments, physiology "will not fail to gain something, and perhaps something considerable, by "its falsehood."

Such, Gentlemen, is the protest upon the authority of which Dr. Beddoes says I have given a false representation of his opinion. Let the rest of the medical world be the jury to give a verdict on this contested point. If they can discover in this passage a formal protest against his entertaining similar speculations with Dr. Girtanner concerning oxygen, I shall then conclude that Dr. Beddoes has had the address to state two opinions which destroy each other, but where I could not see the opposition between them. To me this paragraph appears only to be a kind of apology for a most extravagant extension which he (Dr. Beddoes) has made of the hypothesis, and which immediately precedes the protest.

"Was not Mayow," says Dr. Beddoes, p. 258, "infinitely nearer the "truth, than any author of a later hypothesis, when he imputed muscular "motion to the effervescence of his nitro-atmospherical particles? Does "not muscular contraction or intumescence really depend upon the com- "bination of oxygen with hydrogen (separately, and combined in various "proportions), in consequence of a sort of explosion produced by the "nervous electricity? According to this hypothesis, animal motion, at "least that of animals analogous to man, would *be produced by a very* "*beautiful pneumatic machinery*; and our nervous and muscular systems "may be considered as *a sort of steam-engine*. This hypothesis, though not perhaps

" perhaps at this moment capable of strict proof, *is extremely probable,* since " it is countenanced *by every observation and experiment yet made on the " subject.*"

I hope, it will appear from these passages that I have not given a false representation of Dr. Beddoes's opinions.

Your zeal in communicating whatever concerns the interests and honor of the profession, your impartiality and love of justice, have induced me to present you with this letter, which you will oblige me by inserting in your Journal.

I am, Gentlemen,

With much regard,

Your obedient, humble servant,

ALEXANDER CRICHTON.

No. 15, Clifford-Street,

*15th September,* 1799.

